# Open government data portals in the European Union: A dataset from 2015 to 2017

**DOI:** 10.1016/j.dib.2020.105156

**Published:** 2020-01-21

**Authors:** Susana de Juana-Espinosa, Sergio Luján-Mora

**Affiliations:** a Department of Business Organization, University of Alicante, Spain; b Department of Software and Computing Systems, University of Alicante, Spain

**Keywords:** Open government data (OGD), European union, Socioeconomic indicators, Public administration, Clustering

## Abstract

Open government data (OGD) portals are official websites where governments can publish OGD in a controlled way. OGD portals foster discoverability, accountability, and reusability for stakeholders. This data article presents the data collected while monitoring the OGD portals in the 28 countries of the European Union. Several parameters and indicators observed over a period of 3 years in the official national open data portals were located and recorded to create this dataset. Data were manually obtained from existing public data sources and official OGD portals freely available on the Web. Clustering techniques using Density-based spatial clustering of applications with noise (DBSCAN) were applied to elaborate a dataset showcasing similar countries with respect to different parameters and indicators. Cluster data validation was carried out using the Davies–Bouldin index. The data presented in this article are related to the research article entitled “Open government data portals in the European Union: Considerations, development and expectations” [1].

**Table:** Specifications Table

Subject	Computer Science (General)
Specific subject area	Open Data, Public administration management
Type of data	Table CSV file Excel file
How data were acquired	Manual extraction from public data sources on WWW Manual review of official open government data portals on WWW
Data format	Raw Processed
Parameters for data collection	From public data sources: Corruption Perceptions Index; e-Government Data Index; Employment in high- tech, manufacturing and knowledge-intensive service sectors; Expenditure; Gross Domestic Product; Global Open Data index; Internet connections; Number of patents; Portal Maturity index; Population From official open government data portals: Number of datasets, number of applications available, and number of organizations participating collected from official open government data portals on July 2015, October 2016, and December 2017.
Description of data collection	Data manually collected from public data sources and official open government data portals on WWW; data recorded as Excel files.
Data source location	Data available on the WWW from the 28 countries belonging to the European Union at July 1st, 2015: Austria, Belgium, Bulgaria, Croatia, Cyprus, Czech Republic, Denmark, Estonia, Finland, France, Germany, Greece, Hungary, Ireland, Italy, Latvia, Lithuania, Luxembourg, Malta, Netherlands, Poland, Portugal, Romania, Slovakia, Slovenia, Spain, Sweden, and United Kingdom
Data accessibility	Repository name: Mendeley Data Data identification number: 55y8zdcnbt.1 Direct URL to data: https://doi.org/10.17632/55y8zdcnbt.1
Related research article	de Juana Espinosa, S. & Luján-Mora, S. (2019. “Open government data portals in the European Union: Considerations, development, and expectations”, Technological Forecasting and Social Change, 149, 119769. https://doi.org/10.1016/j.techfore.2019.119769

**Table:** 

**Value of the Data** • To our knowledge, there is no other collection of public datasets on the evolution of the official open government data portals of the 28 countries of the European Union. • The raw data of the evolution of the official open government data portals of the 28 countries of the European Union can be useful to understand the current situation and future trends of open government data in the European Union and abroad. • Data collections on open government data portals should be available for the empowerment of open government data users, researchers and other stakeholders. • The collected data can be used to comprehend the evolution of the official open government data portals of the 28 countries of the European Union and forecast future scenarios. • Policymakers and academic researchers may find this collection of data useful for the formulation, analysis and deployment of more efficient public policies.

## 1 Data

According to the Open Knowledge Foundation [2], open data refers to data that may be “… freely accessed, used, modified, and shared by anyone for any purpose”. In accordance with the Organization for Economic Co-operation and Development, open government data (OGD) is “a philosophy- and increasingly a set of policies - that promotes transparency, accountability and value creation by making government data available to all” [3]. The geopolitical context is a crucial factor in the development of OGD, since the effectiveness of open government policies is influenced by cultural, geographical or regulatory factors tied to the country [4,5]. Open data portals are “web-based interfaces designed to make it easier to find re-useable information” and are “an important element of most open data initiatives” [6].

The dataset presented in this paper was created with the objective of supporting the analysis of the development of OGD portals in the 28 countries of the European Union [1]. The dataset combines the socioeconomic statistics about the countries of the OGD portals and the data about the OGD portals. OGD portals “suffer from the large number of diverse data structures that make the comparison and aggregate analysis of government data practically impossible” [7]. In addition, the lack of a single point of access to the OGD portals makes it difficult to locate and access the open data they provide. However, in order to foster comparability of data published across OGD portals, it is needed to collect and store data in a common format.

The dataset has been published in Mendeley Data.^1^ It comprises 16 Excel files, as described in Table 1.

**Table 1:** Content of the dataset published in Mendeley Data.

Filename	Size (KB)	Number of rows	Description
Data aggregation.xlsx	23	87	Data collected from statistical sources and the official OGD portals of each EU country in July 2015, October 2016 and December 2017
2015 July out2-score.csv	65	608	Clustering of 2 variables from the data collected from official OGD portals of each EU country in July 2015
2015 July out3-score.csv	520	4562	Clustering of 3 variables from the data collected from official OGD portals of each EU country in July 2015
2015 July out4-score.csv	2366	19,753	Clustering of 4 variables from the data collected from official OGD portals of each EU country in July 2015
2015 July out5-score.csv	7006	56,120	Clustering of 5 variables from the data collected from official OGD portals of each EU country in July 2015
2015 July out6-score.csv	14,049	108,412	Clustering of 6 variables from the data collected from official OGD portals of each EU country in July 2015
2016 October out2-score.csv	53	504	Clustering of 2 variables from the data collected from official OGD portals of each EU country in October 2016
2016 October out3-score.csv	414	3736	Clustering of 3 variables from the data collected from official OGD portals of each EU country in October 2016
2016 October out4-score.csv	1870	16,021	Clustering of 4 variables from the data collected from official OGD portals of each EU country in October 2016
2016 October out5-score.csv	5352	43,807	Clustering of 5 variables from the data collected from official OGD portals of each EU country in October 2016
2016 October out6-score.csv	10,733	84,007	Clustering of 6 variables from the data collected from official OGD portals of each EU country in October 2016
2017 December out2-score.csv	48	459	Clustering of 2 variables from the data collected from official OGD portals of each EU country in December 2017
2017 December out3-score.csv	340	3049	Clustering of 3 variables from the data collected from official OGD portals of each EU country in December 2017
2017 December out4-score.csv	1586	13,323	Clustering of 4 variables from the data collected from official OGD portals of each EU country in December 2017
2017 December out5-score.csv	4836	38,355	Clustering of 5 variables from the data collected from official OGD portals of each EU country in December 2017
2017 December out6-score.csv	10,206	76,924	Clustering of 6 variables from the data collected from official OGD portals of each EU country in December 2017

The file “Data aggregation.xlsx” contains the primary dataset composed from 10 public socioeconomic data sources (see Table 2), and 4 indicators gathered from the OGD portals of the 28 countries of the European Union over a period of three years, from July 2015 to December 2017, once every 13 months. The data is presented in the following spreadsheets:

• Variable description: Definition of the variables; it includes the acronym of the variable; its definition and the source of the data (see Table 2).
• 2015 July: Data acquired and collected in July 2015 (see an example of this spreadsheet in Table 5).
• 2016 October: Data acquired and collected in October 2016 (see an example of this spreadsheet in Table 5).
• 2017 December: Data acquired and collected in December 2017 (see an example of this spreadsheet in Table 5).

**Table 2:** Description of data on the file “Data aggregation.xlsx”.

Variable	Type and scale	Description	Source
CPI	Integer, [0, 100]	Corruption Perceptions Index	Transparency International
EGDI	Real, [0, 1]	e-Government Data Index	UN E-Government Knowledgebase – UN E-Government Survey
EHT	Real, [0, 100]	Employment in high- tech, manufacturing and knowledge-intensive service sectors (as % of total employment)	European Commission – Eurostat
EXP	Real, millions of euros	Expenditure	European Commission – Eurostat
GDP	Real, millions of euros	Gross Domestic Product in millions of euros	European Commission – Eurostat
GODI	Integer, [0, 100]	Global Open Data index	Open Knowledge International
IU	Real, [0, 100]	Internet connections (as % over total of population)	International Telecommunication Union
PAT	Real, number per 1 million inhabitants	Number of patents	European Commission – Eurostat
PM	Real, [0, 100]	Portal Maturity index	European Commission – European Data Portal
POP	Integer	Population	European Commission – Eurostat

**Table 3:** URLs of the official open government data portals of the EU countries.

Code	Country	URL of official portal
AT	Austria	https://www.data.gv.at/
BE	Belgium	http://data.gov.be/
BG	Bulgaria	http://opendata.government.bg/
HR	Croatia	http://data.gov.hr/
CY	Cyprus	http://www.data.gov.cy/
CZ	Czech Republic	http://data.gov.cz
DK	Denmark	https://data.digitaliser.dk/
EE	Estonia	https://opendata.riik.ee/
FI	Finland	https://www.avoindata.fi/en
FR	France	https://www.data.gouv.fr/
DE	Germany	https://www.govdata.de/
EL	Greece	http://data.gov.gr/
HU	Hungary	http://kozadat.hu/
IE	Ireland	http://data.gov.ie/
IT	Italy	http://www.dati.gov.it/
LV	Latvia	https://data.gov.lv/lv
LT	Lithuania	Not located
LU	Luxembourg	https://data.public.lu/
MT	Malta	http://data.gov.mt/
NL	Netherlands	https://data.overheid.nl/
PL	Poland	https://danepubliczne.gov.pl/
PT	Portugal	http://www.dados.gov.pt/
RO	Romania	http://data.gov.ro/
SK	Slovakia	http://data.gov.sk/
SI	Slovenia	http://data.gov.si/nio/
ES	Spain	http://datos.gob.es/
SE	Sweden	http://oppnadata.se/
UK	United Kingdom	http://data.gov.uk/

**Table 4:** Data acquired from the official OGD portal of Spain.

Year	2015 July	2016 October	2017 December	Comment
Number of datasets	8493	11,950	16,411	The number of datasets published is available in Data Catalog – Datasets.^a^
Number of organizations	92	99	111	The number of organizations or publishers is not directly available; it has to be manually counted from the list of publishers.
Number of applications	149	163	203	The number of applications published is available in Impact – Applications.^b^

a https://datos.gob.es/en/catalogo.

b https://datos.gob.es/en/aplicaciones.

The files “2015 July outX-score.csv”, “2016 October outX-score.csv”, and “2017 December outX-score.csv”, log the clustering of the data in “Data aggregation.xlsx”, where X ranges from 2 to 6, for all the combinations of X variables from the dataset of the whole group of variables (see Table 6). These files are in the comma-separated values (CSV) format.

**Table 5:** Example of data collected from open government data portals and public data sources.

A	B	C	D	E	F	G	H	I	J	K	L
Country	Population	GDP	EXP	AGE	Number of datasets	Number of organizations	Number of applications	PM	GODI	IU	CPI
BE	11,237,274	411010.2	220913.9	4.58082192	117		10	37.2	43	85.0529	77
BG	7,202,198	45288.5	18332.1	0.27945205	42	29		60.0	56	56.6563	41
HR	4,225,316	44605.9	21538.6	0.36712329	106	27	16	48.0		69.8031	51
CY	847008	17746.0	7212.5	0.67945205	160		1	35.5		71.7159	61
CZ	10,538,275	168473.3	70251.5					20.0	52	75.6688	56

**Table 6:** Structure of clustering files.

A	B	C	D	E … F	G	H	I … Z
number of variables used in the clustering	epsilon	minpoints	DBI	variables used in the clustering; the actual number of columns is equal to (column A)	number of clusters	number of countries	clusters with the codes of the countries grouped in each cluster

## 2 Experimental design, materials, and methods

Extensive online searches and exploring websites of the key organizations were used to identify the public data sources for the socioeconomic statistics about the countries of the OGD portals on the file “Data aggregation.xlsx”. A detailed description of the data collected from the public data sources, including the acronym of the variable, the type and scale of the variable, the description of the variable and the source of the variable, is shown in Table 2. The exact URL of each source is included in the spreadsheet “Variable description” of the file “Data aggregation.xlsx”.

Besides, the following four variables were compiled:

• AGE, the age of the OGD portal, measured in years since its publication.
• The number of datasets published on the OGD portal.
• The number of organizations or publishers announced on the OGD portal.
• The number of applications published on the OGD portal.

These data were collected manually by means of an online review of the official OGD portal of each country. Table 3 shows the URL of the official OGD portals of the EU countries. The official OGD portal of Lithuania could not be located, although thorough searches were conducted.

Table 4 shows the data collected in the case of Spain for the sake of illustration. Its official OGD portal was launched^2^ in October 2011.

Table 5 shows an excerpt of the data collected from OGD portals and public data sources. The file “Data aggregation.xlsx” contains the dataset created before the pre-processing for July 2015, October 2016, and December 2017. Pre-processing includes different tasks such as to identify and correct possible errors (missing values and outlier values), and to normalize all values for effective comparison. The structure of the spreadsheets available in the file “Data aggregation.xlsx” follows the schema illustrated in Table 2.

As it was mentioned before, the files “2015 July outX-score.csv”, “2016 October outX-score.csv”, and “2017 December outX-score.csv” are generated by the clustering of the dataset in the file “Data aggregation.xlsx”. The clustering applies the Density-based spatial clustering of applications with noise (DBSCAN) algorithm [8] which requires two input parameters: a) epsilon, the maximum radius to test the distance between data points; and b) minpoints, the minimum number of points needed to create a cluster. The clustering is iteratively performed with epsilon taking values from 0.5 to 3.0, with steps of 0.1, and minpoints taking integer values from 2 to 7. If the distance between two points is lower or equal to epsilon, then those two points will be in the same cluster. To validate the clusters obtained, the Davies–Bouldin index (DBI) [9] is applied, which basically measures the compactness and separation of the clusters. The selection process applies the following heuristics:

• To maximize the number of portals (countries) grouped in each cluster, to classify as many portals as possible.
• To maximize the number of portals (countries) grouped in each cluster, to make a fine classification of the portals.
• To minimize the DBI, to find natural partitions among the portals.

**Fig. 1:**
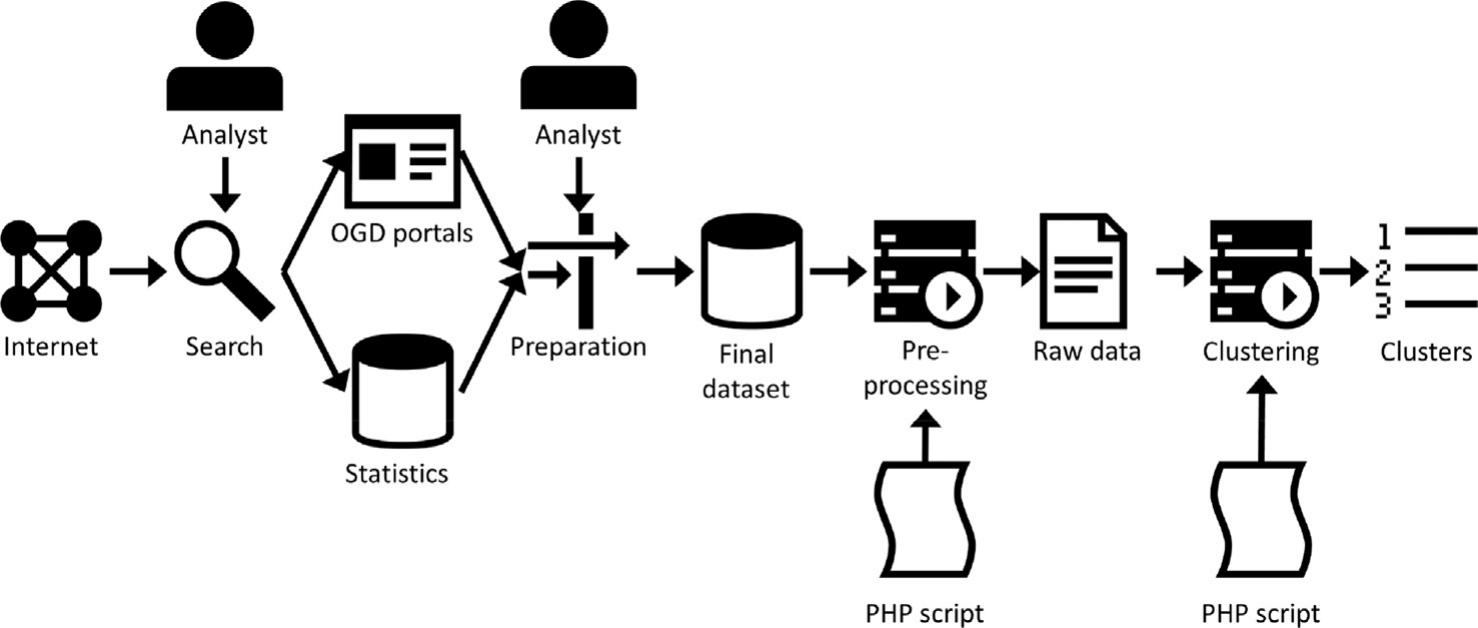
Methodology of creation of the dataset.

The structure of these files follows the schema in Table 6:

To create this dataset, a methodology consisting of 9 steps was carried out, which is visually summarized in Fig. 1:

1. Manual search by an analyst of public data sources with socioeconomic statistics of the 28 countries of the European Union.
2. Manual search by an analyst of official OGD portals of the 28 countries of the European Union.
3. Manual acquisition of data from public data sources.
4. Manual collection of data from official OGD portals
5. Supervised preparation by an analyst of the data aggregated for the final dataset. A data cleaning process prevented the apparition of possible errors in the dataset. The main tasks performed during the data cleaning process were:
  o Transformation of thousands and decimal separators: some systems use the dot “.” as thousands separator and the comma “,” as the decimal separator, whereas other systems do the opposite.
  o Transformation of data formats, e.g., numbers-as-text transformed into real numbers.
6. Automatic pre-processing by a PHP script. The components of the dataset present different dimensions and magnitudes; therefore, there is a need to normalize all components into dimensionless for effective comparison.
7. Automatic clustering by a PHP script. An iterative process calculates all the combinations of k variables from the set of n variables and the clustering divides data into groups with similar values. The clustering applies the DBSCAN algorithm [8].
8. Validation of clustering by the Davies–Bouldin index [9].
9. Creation of the clustering data files.

## Authors' contributions

All the authors have contributed equally to collecting, analyzing and discussing the data and to writing this article. All the authors have read and approved this article.

## Footnotes

1 https://doi.org/10.17632/55y8zdcnbt.1.

2 https://datos.gob.es/es/noticia/datosgobes-inicia-sus-pasos-en-beta.
